# The impact of childhood maltreatment on treatment outcomes for posttraumatic stress symptoms and aggression in male former combatants using narrative exposure therapy [NET] - results from a RCT in Eastern democratic Republic of Congo

**DOI:** 10.1186/s13031-025-00710-z

**Published:** 2025-10-04

**Authors:** Tobias Rieder, Katy Robjant, Amani Chibashimba, Sabine Schmitt, Stephan Goerigk, Thomas Elbert, Anke Koebach, Andrea Jobst

**Affiliations:** 1 Teaching and Training Unit, Division of Infectious Diseases and Tropical Medicine, LMU University Hospital Munich, Munich, Germany; 2 Department of Psychology, University of Konstanz, Konstanz, Germany; 3 Nongovernment organization vivo international e.V., Konstanz, Germany; 4 Charlotte Fresenius University Munich, University of Psychology, Munich, Germany; 5 Department of Psychiatry and Psychotherapy, LMU University Hospital, Munich, Germany; 6 Center for International Health, LMU University Hospital, Munich, Germany; 7 University of the Bundeswehr Munich, Institute of Psychology, Munich, Germany

## Abstract

**Objective:**

This study investigates the impact of childhood maltreatment on treatment outcomes among male ex-combatants in a randomized controlled trial (RCT) of Narrative Exposure Therapy for Forensic Offender Rehabilitation (FORNET), a specialized psychotherapy used to treat trauma sequelae including symptoms of posttraumatic stress disorder (PTSD), compared with treatment as usual (TAU). Specifically, we aim to compare former child and adult male soldiers who experienced childhood sexual abuse (CSA) with those who did not.

**Methods:**

We conducted a sub-analysis of data from Koebach et al. [J Consult Clin Psychol. 2021], focusing on a sample of male former soldiers in the eastern Democratic Republic of Congo (DRC). Participants were categorized into two groups based on their history of CSA. Outcome measures included the prevalence of lifetime sexual assaults, perpetration of sexual violence against others, appetitive aggression, current violent behavior, symptoms of PTSD and depression and responses to two treatment modalities: TAU and FORNET.

**Results:**

The group with a history of CSA had significantly higher rates of re-experiencing sexually assaults, especially by superiors, and of perpetrating sexual assaults against others. In addition, this group presented elevated baseline scores in all outcomes (appetitive aggression, current violent behavior, symptoms of PTSD and depression). Regarding effectiveness of treatment arms, the FORNET group demonstrated significantly greater reductions in appetitive aggression levels, PTSD symptoms and depressive symptoms compared to the TAU group, with no difference in treatment effectiveness between participants with and without a history of CSA. However, individuals with CSA showed statistically superior improvements in current violent behavior, with similar score levels to those without CSA after 6–9 months.

**Conclusion:**

CSA among former soldiers was significantly associated with a higher prevalence of PTSD and increased risk of both sexual revictimization and the perpetration of sexual and other violent acts. FORNET demonstrates effectiveness in reducing appetitive aggression, PTSD symptoms, and violent behavior even in the subgroup highly affected by CSA - showing an even greater impact on current violent behavior. The ability of NET to address trauma and perpetration in a chronological sequence and adapt to the specific challenges of CSA likely account for its effectiveness in treating this complexly traumatized population, ultimately contributing to a reduction of violence in post-conflict communities. Special attention should be paid to revictimization during the rehabilitation process of ex-combatants.

**Supplementary Information:**

The online version contains supplementary material available at 10.1186/s13031-025-00710-z.

## Introduction

Trauma is a pervasive vulnerability affecting millions worldwide, often leading to severe mental health problems including the development of trauma spectrum disorders, such as posttraumatic stress disorder (PTSD). Trauma can result from various sources, including warfare, sexual violence (as a victim and in rare cases from perpetration of violent acts), and childhood abuse [48]. Members of armed groups, including criminal gangs, are at particularly high risk of both exposure to traumatic experiences and perpetration of violent acts. Studies from various conflict regions around the world reveal the profound impact of war-related, interpersonal trauma exposure, as well as acts of perpetration, on mental health and social functioning, with reports of posttraumatic stress symptoms [31, 34, 42, 65] and heightened levels of aggression [6, 41, 60, 62, 64]. These can lead to increased violence in families and communities, affecting societies at large, particularly in post-conflict settings or states with poor governmental control [41, 46, 54].

Trauma that occurs during the vulnerable phase of childhood appears to have the most detrimental effect on mental health outcomes later in life, especially after subsequent traumatization and constitutes a risk factor for mental health problems and disorders [4, 36]. The concept of cumulative trauma load posits that the accumulation of traumatic experiences over lifetime exacerbates the risk and severity of PTSD and other mental disorders [20]. Thereby, experiencing trauma and maltreatment during sensitive periods of childhood might play a crucial role for an increased vulnerability to further potential lifetime stressors and traumatic life events with higher probability for the development of trauma spectrum disorders and chronic PTSD [38, 44] Studies have shown that individuals with a history of childhood sexual abuse (CSA) are more likely to suffer from PTSD after a new traumatic event compared to those without such a history [8]. This increased risk is often attributed to the development of maladaptive coping mechanisms and a heightened sensitivity to stressors. Moreover, among people with mental disorders, a history of childhood maltreatment has been found to be associated with greater symptom severity, more pronounced functional impairments, poorer quality of life, and chronic course of disease** (**Medeiros et al., [39, 40, 52, 53]. Additionally, individuals who experience abuse in childhood are at higher risk for revictimization (Fereidooni, 2023). Indeed, particularly CSA can lead to a persistent victim mode, characterized by heightened vulnerability and a tendency to experience further (sexual) victimization [63]; [19, 57, 58]. Furthermore, childhood maltreatment has been associated with later violent, aggressive [59] and antisocial behavior (Braga et al.) [7], especially when it includes violent acts. This includes the development of appetitive aggression - a form of aggression experienced as rewarding or pleasurable - which was particularly elevated in these populations.

The phenomenon of appetitive aggression is especially relevant in the context of former combatants or soldiers, where early exposure to violence may predispose individuals to aggressive behaviors later in life (Hecker, Hermenau, Maedl, Schauer, et al.,) [25]. Generally, the motives for aggression can be divided into reactive aggression, proactive (predatory) aggression, and appetitive aggression [13, 14]. Reactive aggression is driven by angry emotions in defense or as a response to perceived threats. Proactive aggression is goal-oriented, aiming to obtain an advantage such as material goods or improved social status [2, 14]. Appetitive aggression, on the other hand, involves the intrinsically rewarding experience of aggressive action, eliciting excitement and pleasure rather than fear or terror [13, 14]. This form of aggression has addictive potential, as it activates the brain’s reward system—specifically, dopaminergic pathways such as those involving the nucleus accumbens, which are associated with pleasure, motivation, and reinforcement **(**Golden et al., [23, 45]. Elbert et al. [13] have reconciled the dissonance between aversive traumatic experiences and lustful perpetrated acts by formulating bi-cycles of violence. In this model, traumatization, posttraumatic stress symptoms, and reactive aggression on the one hand, and perpetration of violence, appetitive aggression, pro-violent norms, and proactive perpetration on the other, accelerate each other. In post-conflict settings, appetitive aggression perpetuates the cycle of violence, ultimately leading to increased violence in families and the community, including gender-based and intimate-partner violence and crimes. Therefore, treatment opportunities, which address both, traumatic stress symptoms and appetitive aggression, are essential to break the cycle. To this end, FORNET, an adaptation of Narrative Exposure Therapy (NET) for Forensic Offender Rehabilitation, has been specifically invented to treat the sequelae of perpetration and appetitive aggression alongside victimization and PTSD symptoms [12]. NET is a short-term therapeutic intervention designed to help individuals process and integrate traumatic memories by constructing a coherent narrative of their experiences [50]. The original version of NET has been particularly effective in treating PTSD among populations exposed to severe and prolonged trauma, such as refugees and former combatants or soldiers [43]. Regarding FORNET, studies have found improvement in PTSD symptoms [27, 29, 34] and aggressive behavior, (e.g., physical abuse against children, intimate partner and community violence) [10, 28] without loss in effectiveness when delivered by trained local non-health professionals (Koebach et al.) [56]. FORNET has also shown promising results for the treatment of substance use disorders (Koebach et al.,) [56]. A large RCT on FORNET, conducted in the Eastern Democratic Republic of Congo (DRC) from 2016 to 2019 and comprising 448 former male soldiers, demonstrated the superiority of FORNET—performed in parallel individual and group sessions—over treatment as usual (TAU) in improving appetitive aggression, current violent behavior (CBV; i.e., perpetrated acts against intimate partners, own children or others), and PTSD symptomatology over a 6-month follow-up period [33]. The study utilized a cascade training (train-the-trainer) approach. In summary, FORNET has the potential to support demobilization -formal discharge and reentry of combatants into civilian life -, social reintegration and peacebuilding efforts within post-conflict societies.

However, childhood maltreatment might be a relevant factor influencing psychotherapy outcomes. For instance, a meta-analysis of treatment outcomes in depression revealed that a history of childhood maltreatment predicted unfavorable treatment outcomes** (**Nanni et al., [40]). A very recent re-analysis of a randomized controlled trial comparing effectiveness of CBASP and non-specific (supportive) psychotherapy in 253 adults with early-onset chronic depression reveals that the effect of psychotherapy highly depends on distinct clusters of childhood maltreatment, with CBASP showing superiority for particularly severe trauma profiles [22, 51]. Regarding PTSD treatment outcomes, individuals with a history of CSA may exhibit higher levels of PTSD symptoms, which can complicate treatment. These findings underscore the importance of addressing CSA in therapeutic settings to improve long-term outcomes [56].

So far, the effectiveness of Narrative Exposure Therapy (NET) or its adapted version FORNET has not been systematically analyzed regarding the potential influence of CSA on treatment outcomes. Therefore, in this investigation, we performed a retrospective sub-analysis of the large data set, from the aforementioned RCT by Koebach et al. [33], conducted in the DRC, which compared short-term FORNET and TAU. We specifically focus on CSA among other types of childhood maltreatment due to its high relevance for subsequent sexual revictimization and perpetration in the context of combat-trauma.

## Methods

### Study design

The RCT on treatment outcomes in PTSD-affected former soldiers from the Kivu regions of Eastern DRC was conducted from October 2016 until April 2019. Details were previously described [33]. The RCT comprised a randomized, assessor-blind, parallel group design to evaluate the effectiveness of Narrative Exposure Therapy for Forensic Offender Rehabilitation (FORNET), which consisted of six weekly parallel individual and group sessions, versus treatment as usual (TAU). TAU clients continued to receive the support services provided by NGOs, such as occupational training courses, empathic listening, problem-solving, and practical support upon request. Assessment visits included the baseline measurement, a post-intervention measurement, as well as follow-up investigations at 3–5 months and 6–9 months. Structured clinical interviews were conducted by seven Congolese psychological interviewers trained in informed consent procedures, standardized assessment tools, empathic interviewing, and RCT protocols, including blinding and confidentiality. They received supervision and live observation, and blinding was maintained by instructing participants not to reveal whether they had met a counselor, which would indicate FORNET participation. The trial was conducted in Goma, the provincial capital of North Kivu, Eastern DRC. This region has experienced prolonged conflict, making it a pertinent location for studying former combatants and soldiers and the humanitarian need. The need for specific trauma-informed interventions, reconciliation work, and peace building efforts, has been high in the region.

Participants were enlisted through local NGOs providing services to former combatants. Interviewers remained blind to the participants’ group assignments. Baseline interviews were conducted in six two-month periods, creating clusters for a stratified block randomization based on the severity of PTSD and appetitive aggression (for more detail see Koebach et al. [33]). Former combatants who agreed to participate gave written consent before the start of the interviews.

#### Ethical approval

Ethical approval for the RCT study was granted by the ethical commission of the University of Konstanz (31/2016) and the Social Fund of the DRC. Additionally, for this secondary analysis, we obtained ethics approval from the ethics commission of the LMU Munich (24–0411).

## Participants

The original intention-to-treat sample included 448 male former combatants aged 16 years up or older. Out of these, 397 (89%) received treatment per protocol. Among those, 394 had information about CSA and were included in the current analysis. Inclusion criteria were (1) at least one traumatic event on a life-event checklist, (2) exhibition of significant PTSD symptoms as diagnosed by DSM-5 criteria, and/or (3) a score greater than 21 on the Appetitive Aggression Scale (AAS; Weierstall et al.,) [61]. Exclusion criteria included (1) acute intoxication (from alcohol or other substances), (2) severe psychotic symptoms, or signs of organic diseases. The age of participants ranged from 16 to 75 years, with an average age of 33.77 years (SD = 8.82), 55 (12.28%) participants were below the age of 18 (16–18). The majority of participants were literate (*n* = 337, 86%) and had an average of 7.99 years of formal education (SD = 4.03). There were no significant differences in these demographics between the intervention and control group (Table 1). For the secondary data analysis, we divided the sample into a group that experienced CSA and a group which did not, based on the answers in the life-event-checklist. Differences in demographics between the CSA (*n* = 47) and no CSA group (*n* = 347) are presented in Table 2.

**Table 1:** Sample characteristics at baseline

Variable	TAU (*n* = 223)	FORNET (*n* = 171)	Total (*n* = 394)
Age, mean (SD), n	33.68 (9.04), 223	33.89 (8.53), 171	33.77 (8.82), 394
Age < 18%, n	3.1, 7	2.3, 4	2.8, 11
Reading and writing literacy, %, n	84.2, 186	88.3, 151	86.0, 337
Education, y, mean (SD), n	7.98 (4.05), 222	8.01 (4.00), 169	7.99 (4.03), 391
Relationship			
Single, %, n	27.5, 61	28.1, 48	27.7, 109
Married/partnership, %, n	48.6, 108	54.4, 93	51.1, 201
Divorced, %, n	21.2, 47	16.4, 28	19.1, 75
Widowed, %, n	2.7, 6	1.2, 2	2.0, 8
Children, mean (SD), n	3.04 (3.06), 223	2.88 (2.60), 170	2.97 (2.87), 393
Child maltreatment			
Neglect, %, n	30.1, 65	26.5, 44	28.5, 109
Sexual abuse, %, n	13.0, 29	10.5, 18	11.9, 47
Verbal abuse, %, n	79.7, 177	87.1, 149	83.0, 326
Hit by parent, %, n	78.0, 174	83.0, 142	80.2, 316
Burnt by parent, %, n	15.2, 34	17.6, 30	16.3, 64
Overall child maltreatment exposure, %, n	88.3, 197	93.0, 159	90.4, 356
Age at military enrolment, mean (SD), n	17.87 (5.81), 215	18.02 (5.81), 168	17.94 (5.81), 383
Joined military voluntarily, %, n	65.3, 145	59.4, 101	62.8, 246
Combat experience, %, n	98.6, 213	99.4, 165	99.0, 378
Perpetrated violence and re-victimization			
Sexually assaulted, %, n	16.6, 37	15.8, 27	16.2, 64
Witnessed sexual assault, %, n	62.3, 139	59.6, 102	61.2, 241
Assaulted by superior, %, n	19.6, 43	16.2, 27	18.1, 70
Sexually assaulted other, %, n	43.7, 97	42.9, 73	43.4, 170

TAU treatment as usual; FORNET narrative exposure therapy for forensic offender rehabilitation; SD standard deviation

**Table 2:** Sample characteristics at baseline CSA vs. no CSA

Variable	No CSA (*n* = 347)	CSA (*n* = 47)	Total (*n* = 394)
Age mean (SD), n	33.57 (8.71), 347	35.26 (9.54), 47	33.77 (8.82), 394
Younger than 18 years, %, n	2.6, 9	4.3, 2	2.8, 11
Reading and writing ability, %, n	85.5, 295	89.4, 42	86.0, 337
Years of education mean (SD), n	7.91 (4.05), 346	8.60 (3.80), 45	7.99 (4.03), 391
Relationship status: Single, %, n	29.5, 102	14.9, 7	27.7, 109
Relationship status: Married/in partnership, %, n	50.9, 176	53.2, 25	51.1, 201
Relationship status: Divorced, %, n	17.6, 61	29.8, 14	19.1, 75
Relationship status: Widowed, %, n	2.0, 7	2.1, 1	2.0, 8
Number of children, mean (SD), n	2.90 (2.64), 347	3.50 (4.19), 46	2.97 (2.87), 393
Received other treatment, %, n	54.2, 129	60.0, 18	54.9, 147
Child maltreatment			
Neglect, %, n	25.0, 85	57.1, 24	28.5, 109
Sexual abuse [CSA], %, n	0.0, 0	100.0, 47	11.9, 47
Verbal Abuse], %, n	82.1, 284	89.4, 42	83.0, 326
Hit by parent, %, n	79.5, 276	85.1, 40	80.2, 316
Burnt by parent, %, n	14.2, 49	31.9, 15	16.3, 64
Overall child maltreatment exposure, %, n	89.0, 309	100.0, 47	90.4, 356
Mean age entry in military (SD), n	17.95 (5.54), 336	17.87 (7.49), 47	17.94 (5.81), 383
Entered military voluntarily, %, n	62.9, 217	61.7, 29	62.8, 246
Combat experience, %, n	98.8, 336	100.0, 42	99.0, 378

CSA childhood sexual abuse

## Assessment

Exposure to trauma, violence and perpetration was assessed at baseline using a 44-item traumatic life event checklist (experience or witnessing of events, dichotomous answers [yes/no]), which had been previously adapted from studies with similar populations in DRC [33]. Seven items ask specifically about childhood maltreatment, of which we used five relevant exposure items. We derived information about the history of childhood maltreatment (items are listed in Table 2 under “Child maltreatment”) and specifically about CSA (CSA item: “Victim of sexual abuse during childhood”) from this answers. The remaining items addressed lifetime exposure to violence and traumatic life events (17 items) as well as perpetrated acts of violence (20 items). For the current investigation, lifetime sexual victimization (being sexually assaulted, beeing sexually assaulted by a superior and witnessed sexual aggression) and the perpetration of sexual violence against others were taken from the four index items, respectively (Table 1).

Inclination towards aggressiveness was measured using the Appetitive Aggression Scale (ASS), a 15-item structured interview assessing the extent of agreement to statements on a 5-point scale (0 = disagree to 4 = agree). The sum score indicates the intensity of appetitive aggression, with a maximum of 60. A median cut-off score for heightened appetitive aggression (< 21) was derived from previous studies with former combatants in East Africa [33]. The AAS has been validated in similar contexts and demonstrated high internal consistency (Cronbach’s α = 0.88) and interrater reliability (IRR = 0.98).

PTSD severity was assessed using the PTSD Symptom Scale Interview for DSM-5 (PSSI-5) [17, 18]. This scale allows for diagnosing PTSD according to DSM-5 criteria (American Psychiatric Association, 2013). Scores are calculated for each of the 20 items by summing responses across clusters B to E, yielding a maximum score of 80. Participants answered with reference to their experiences over the previous month. The DSM-IV version of this instrument has been validated in similar East African settings [15] and the current version shows high consistency in Congolese former combatants [49]. In this study, interrater reliability and internal consistency were highly satisfactory (ICC = 0.94, α = 0.93).

The severity of depressive symptoms was measured using the Patient Health Questionnaire depression module (PHQ-9) [35]. This nine-item questionnaire is scored from 0 (not at all) to 3 (nearly every day) based on symptom presence in the previous two weeks, with a maximum sum score of 27. Cut-off points for mild, moderate, moderately severe, and severe depression are 5, 10, 15, and 20, respectively [35]. Reliability outcomes in our sample were satisfactory and comparable to previous reports from Africa [9, 21], with ICC = 0.94 and α = 0.86.

Current violent behavior (CVB) perpetrated in the past three months was measured using a 32-item checklist of aggressive acts against intimate partners, own children (if living in the household), or others, with dichotomous response options (yes/no). Sum scores, corrected for whether the participant had children or an intimate partner, provided a measure of CVB ranging from 0 to 32. The CVB checklist has been used in studies with former combatants in Burundi [3, 11] and female former child soldiers in the DRC [47]. The internal consistency and interrater reliability were highly satisfactory (α = 0.97, Cohen’s κ = 0.93).

## Intervention

FORNET, originally introduced by Elbert et al. [12], has been implemented in Eastern DRC [27]; Koebach et al., [49], Burundi [11], and South Africa [28, 29] with slight variations in session numbers and group components. In this study, FORNET was administered over six weekly individual and parallel group sessions, each lasting 90–120 min. The initial individual session involved creating a lifeline to chronologically map out traumatic events, perpetrated acts, and positive experiences. The lifeline is a visual timeline of the participant`s life, used to sequence traumatic and perpetrated events. It is symbolized by a rope, with stones representing traumatic events, sticks for acts of perpetration, and flowers for positive experiences. Based on this lifeline and the selection principles outlined in Robjant et al. [47], a plan for the subsequent five exposure sessions was developed. Following NET methodology, which involves processing all lifetime trauma in chronological order, we assumed that if childhood trauma was present, it would have been processed during FORNET in the sequence of traumatic events, alongside all trauma experienced later in life, including trauma associated with the current conflict.

The first group session focused on transitioning from armed group membership to civilian life, mirroring other implementations [27, 34]. The second and third sessions provided psychoeducation and taught basic skills in anger recognition, emotion regulation, and behavioral change to avoid violence. A “buddy system” was introduced in the second session, encouraging clients to pair up for mutual support in managing frustrations and resolving conflicts non-violently. Sessions three to six involved deconstructing recent experiences of violence and avoiding violent behavior over the previous week. Therapists gradually decreased their active role over the sessions. Clients were encouraged to continue meeting after the group sessions ended. Each group consisted of four clients [47]. FORNET was delivered by Congolese personnel who underwent a three-week training program and were familiar with the local language and circumstances. These individuals had higher education backgrounds but no prior formal mental health training. The initial training was led by a clinical psychologist and later by trained Congolese FORNET trainers under supervision. The training focused heavily on developing clinical skills for exposing traumatic/perpetrated events and conducting group sessions.

The treatment as usual (TAU) group continued receiving services from their respective NGOs, which included occupational training courses, empathic listening, problem-solving, and practical support upon request. Many TAU clients continued to visit the NGOs where they were recruited, engaging in activities such as sports and games, and receiving practical advice and social support from peers.

### Statistical analysis

The effect of the history of CSA on dichotomous outcomes (i.e., lifetime sexual victimization, perpetration, PTSD diagnosis after 6–9 months [no/yes]) was evaluated using logistic regression models. Baseline differences in levels of continuous outcomes (i.e., PTSD symptom severity, depressive symptom severity, Appetitive Aggression (AAS) and current violent behavior [CVB]) by CSA history were tested using linear regression models. Change in continuous outcomes was evaluated using linear mixed models (LMM), which included a continuous time factor (baseline, 3–5 months, 6–9 months) assuming linear change, group (TAU vs. FORNET), and their interaction as fixed effects. Measurements were considered as nested within patients. Significance of model factors was evaluated using type-III F-tests. Estimates were computed using restricted maximum likelihood estimation and the Kenward-Roger method to adjust degrees of freedom. All regression models were adjusted for age by including it as a covariate. Cross-sectional analyses used complete cases and data for longitudinal analyses was not imputed because LMMs can handle missing values and unbalanced distributions. Statistical analyses were performed in R, version 4.4.1. Results were considered statistically significant at $\alpha$=0.05. Effect sizes were reported as Cohen’s *d* using the emmeans::eff_size function.

## Results

Out of the 397 male patients who received treatment per protocol, 394 (99%) had information about CSA and were included in subsequent analyses (TAU: 223 [57%], FORNET: 171 [43%]). Out of these, 47 (12%) reported a history of CSA (TAU: 29 [13%], FORNET: 18 [11%]). Participants were mean (SD) 33.77 (8.81) years old. Baseline sample characteristics are summarized in Tables 1 and 2.

## Pre-treatment differences of participants with and without childhood sexual abuse

**Fig. 1:**
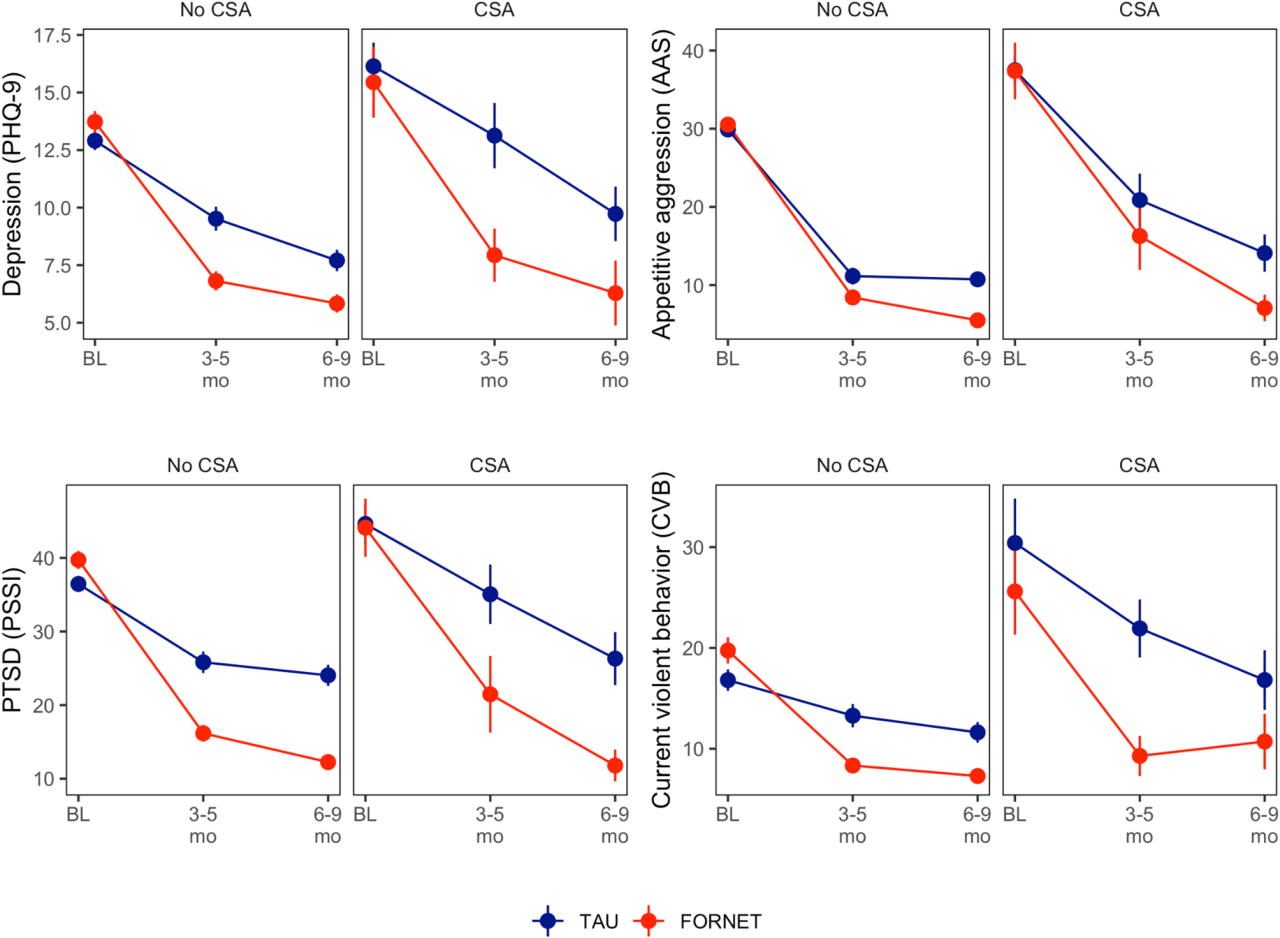
Change in treatment outcomes by treatment group and CSA

Participants with CSA exhibited higher severity of PTSD symptoms (PSSI: 44.34 vs. 37.92, *p* =.007, Cohen’s *d* = − 0.43), depression symptoms (PHQ-9: 15.73 vs. 13.29, *p* =.006, Cohen’s *d* = − 0.45), appetitive aggression (AAS: 37.46 vs. 30.17, *p* <.001, Cohen’s *d* = − 0.63), and current violent behavior (CVB: 28.44 vs. 18.13, *p* =.001, Cohen’s *d* = − 0.64) compared to those without CSA (Fig. 1; Table 3). Distribution of other types of childhood maltreatment appeared similar between the CSA and noCSA group, but with extremely high prevalence of 83% (noCSA group) and 89% (CSA group) emotional abuse and 78% (noCSA group) and 88% (CSA group) physical abuse (Table 2).

## Association of childhood sexual abuse with perpetrated violence and re-victimization

Participants with a history of CSA were more likely to perpetrate violence (OR = 2.48, CI_95%_=1.33 to 4.77, *p* =.005) and showed significantly higher revictimization in various forms, including being sexually assaulted, (OR = 4.06, CI_95%_=2.06 to 7.86, *p* <.001), being sexually assaulted by a superior (OR = 4.16, CI_95%_=2.12 to 8.04, *p* <.001) and witnessing sexual aggression (OR = 2.27, CI_95%_= 1.15 to 4.81, *p* =.024) compared to those without such a history (Table 1).

### Differential efficacy of FORNET vs. TAU

We found significant time effects (all *p* <.05) indicating participants’ improvement on all outcomes across treatment groups, including patients with and without CSA. Patients who received FORNET showed statistically superior improvement in depressive symptoms (F_(1,625)_ = 7.41; *p* =.007), PTSD symptoms (F_(1,618)_ = 23.13; *p* <.001), and appetitive aggression (F_(1,642)_ = 5.69; *p* =.017) and superior improvement in current violent behavior just missing significance (F_(1,612)_ = 3.18; *p* =.075). While participants with a history of CSA showed elevated baseline scores in all outcomes, they showed improvements with a significant time x CSA interaction for current violent behavior (F_(1,611)_ = 4.54; *p* =.034) exhibiting similar score levels as the patients without CSA after 6–9 months. The same pattern was observable for PTSD symptoms and appetitive aggression with two-way interactions being just shy of significance (PSSI: F_(1,618)=_3.37; *p* =.067; for AAS: F_(1,641)_ = 2.93; *p* =.087). No significant three-way interactions were observed, indicating no differential treatment effects of FORNET vs. TAU, based on the history of CSA (Fig. 1; Table 4).

Significantly less patients still showed criteria for a PTSD diagnosis after 6–9 months in FORNET compared to TAU ($\:{\chi\:}_{1}^{2}$=16.04, *p* <.001), but no significant group x CSA interaction was detected ($\:{\chi\:}_{1}^{2}$=0.94, *p* =.33) (Supplementary Table 1).

Due to the imbalanced sizes of groups with and without history of CSA (i.e., 47 vs. 347), we estimated that the current sample size would have been sensitive to detect an effect of *d* = 0.43 with a power (1-β) of 80% assuming a two-tailed test with $\:\alpha\:$=0.05.

**Table 3:** Baseline differences in outcomes by CSA

Outcome	Mean (CI_95%_)	CSA	Difference (CI_95%_)	*P* value	Cohen’s d (CI_95%_)
PHQ-9	13.29 (12.68 to 13.89)	No CSA	−2.44 (−4.19 to −0.69)	0.006**	−0.45 (−0.76 to −0.14)
	15.73 (14.09 to 17.37)	CSA			
AAS	30.17 (28.96 to 31.39)	No CSA	−7.29 (−10.81 to −3.77)	< 0.001***	−0.63 (−0.94 to −0.32)
	37.46 (34.16 to 40.77)	CSA			
PSSI	37.92 (36.3 to 39.53)	No CSA	−6.42 (−11.1 to −1.75)	0.007**	−0.43 (−0.73 to −0.12)
	44.34 (39.95 to 48.73)	CSA			
CVB	18.13 (16.4 to 19.85)	No CSA	−10.32 (−15.3 to −5.33)	< 0.001***	−0.64 (−0.95 to −0.33)
	28.44 (23.76 to 33.12)	CSA			

P values determined using linear regression adjusted for age; * *p* <.05, ** *p* <.01, *** *p* <.001

**Table 4:** Change in treatment outcomes by treatment group and CSA

		Change (CI_95%_)			Time x Group			Time x CSA			Time x Group x CSA			
**Outcome**	**CSA**	**FORNET**	**TAU**	**ES** ^†^	**Df**	**F**	**P**	**Df**	**F**	**P**	**Df**	**F**	**P**	**ES (CI**_**95%**_**)**^††^
PHQ-9	No CSA	−7.98 (−9.03 to −6.93)	−5.23 (−6.28 to −4.19)	0.37 (0.08 to 0.28)	1,625.69	7.41	0.007**	1,625.73	1.71	0.191	1,625.75	0.05	0.820	−0.03 (−0.29 to 0.23)
	CSA	−9.67 (−12.85 to −6.5)	−6.42 (−8.96 to −3.89)	0.43 (−0.05 to 0.49)										
AAS	No CSA	−25.4 (−27.92 to −22.88)	−20.03 (−22.5 to −17.55)	0.36 (0.06 to 0.3)	1,641.68	5.68	0.017*	1,641.72	2.93	0.087	1,641.69	0.11	0.737	−0.11 (−0.73 to 0.51)
	CSA	−30.78 (−38.36 to −23.19)	−23.64 (−29.67 to −17.61)	0.48 (−0.09 to 0.57)										
PSSI	No CSA	−27.74 (−30.76 to −24.71)	−12.71 (−15.72 to −9.71)	0.72 (0.26 to 0.46)	1,618.46	23.13	< 0.001***	1,618.51	3.37	0.067	1,618.51	0.00	0.948	−0.02 (−0.77 to 0.72)
	CSA	−33.76 (−42.89 to −24.62)	−18.32 (−25.6 to −11.03)	0.74 (0.09 to 0.65)										
CVB	No CSA	−12.78 (−15.38 to −10.18)	−5.43 (−8.02 to −2.84)	0.41 (0.1 to 0.31)	1,611.88	3.18	0.075	1,611.99	4.53	0.034*	1,611.96	0.83	0.361	0.3 (−0.34 to 0.94)
	CSA	−16.09 (−23.96 to −8.22)	−13.72 (−19.98 to −7.46)	0.13 (−0.21 to 0.35)										

CSA childhood sexual abuse; FORNET narrative exposure therapy for forensic offender rehabilitation; TAU treatment as usual; Df degrees of freedom using Kenward-Roger method; F test statistic on F-distribution; Change for continuous outcomes estimated by multiplying the model slope by 2 (number of measurements beyond baseline)

† Cohen’s* d* for estimated group difference in change from baseline until 6–9 months

††Cohen’s *d* for estimated difference in group differences in change from baseline until 6–9 months

Cohen’s conventions for effect size interpretation: |>.2| small effect, |>.5| medium effect, |>.8| large effect

* *p* <.05, ** *p* <.01, *** *p* <.001

## Discussion

The current retrospective sub-analysis builds upon the large RCT conducted in the DRC by Koebach et al. [33], which established the efficacy of FORNET, a brief, manualized, and scalable psychotherapeutic method, in improving a wide range of mental health and social outcomes in adult former combatants and child soldiers. Koebach et al. demonstrated that FORNET significantly reduced current violent behavior (CVB) towards children, intimate partners, and others, as well as appetitive aggression—both of which were primary outcomes. Furthermore, FORNET effectively alleviated posttraumatic stress symptoms, a secondary outcome, and led to notable improvements in depression, perceived social acknowledgment, and distancing from (para)military life, indicating the intervention’s broad, positive impact. These findings align with earlier studies, such as the RCT by Robjant et al. [47] involving female former child soldiers, underscoring the critical need for effective interventions like FORNET in post-conflict settings to restore mental health and support the reintegration process. Notably, treatment adherence in Koebach et al.‘s study was remarkably high, with a dropout rate of only 4%, demonstrating the feasibility and acceptability of FORNET among participants. Our sub-analysis specifically focuses on the impact of childhood sexual abuse (CSA) and reveals a more nuanced picture. The findings show that even in cases involving CSA, FORNET is able to yield significant positive effects, leading to even greater improvements in current violent behavior, appetitive aggression, and PTSD symptoms compared to cases without CSA.

We identified 47 participants with a history of sexual abuse in childhood in the overall RCT sample. Male former soldiers who experienced CSA exhibited higher levels of PTSD, depression, appetitive aggression and current violent behavior pre-treatment compared to those without such a history (Table 1). This aligns with the hypothesis that CSA accumulates with further life-threatening events, exacerbates mental health issues later in life [20, 63] and has a profound impact on mental health outcomes, which was evident in the findings of this sub-analysis. Additionally, the likelihood of becoming a perpetrator of sexual violence was higher among those who experienced CSA, supporting the notion that early violent experiences can predispose individuals to aggressive behaviors [24] and, when combined with the experience of combatant life and committing violent acts, appetitive aggression. This transition from victim to perpetrator illustrates the cyclical nature of violence, showing how early trauma can shape later behavior. This pattern highlights the urgent need for early interventions that address both the immediate and long-term consequences of abuse. By intervening early, it is possible to disrupt this cycle, reducing the likelihood of future victimization and perpetration, and promoting healthier, more adaptive outcomes for those affected by such trauma. Furthermore, we found that former combatants with a history of CSA showed a higher rate of revictimization, including a greater likelihood of being sexually assaulted themselves, especially by a superior. This increased risk might be attributed to maladaptive coping mechanisms and heightened sensitivity to stressors developed from early trauma [8]. Previous research has shown that survivors of childhood sexual trauma often experience chronic hyperarousal and emotional dysregulation, contributing to persistent PTSD symptoms and a heightened vulnerability to further victimization [37]. Moreover, CSA has been linked to increased rates of substance abuse and self-harming behaviors as maladaptive coping strategies [32]. Regarding other types of childhood maltreatment in our dataset, the prevalence of emotional (83%) and physical abuse (80.2%) in childhood appeared extremely high in the overall sample, possibly reflecting a common social acceptance of distinct principles of parenting and child handling in this local setting and a possibly high pre-war prevalence of such, which together with cumulative conflict associated trauma might have exaggerated the risk and prevalence for PTSD in the region. Compared to American and European samples, the prevalence of neglect versus physical abuse in our dataset from the Congo is skewed, underscoring the need for caution when generalizing findings on childhood maltreatment across cultures. Those with and without a history of CSA in our dataset showed a similar pattern of these additional types of childhood maltreatment (emotional abuse, emotional neglect, physical abuse), highlighting sexual abuse as a target condition for this sub-analysis (Table 1).

FORNET demonstrated superior improvement compared to TAU in reducing PTSD and depressive symptoms as well as appetitive aggression over time for both individuals with and without a history of CSA, with medium effect sizes. This is particularly remarkable, given that only six sessions were conducted by local counsellors. While those with CSA exhibited higher baseline PTSD symptoms, the reduction was substantial across all participants, with the FORNET group showing particularly pronounced improvements. This pattern of effectiveness extended to aggression as well, where FORNET led to a significantly stronger reduction in aggression levels. The consistency of these results across both groups underscores FORNET’s broad effectiveness in managing aggressive behaviors and PTSD, irrespective of CSA history. Similarly, FORNET had a notable impact on depression, with participants in both CSA and non-CSA groups experiencing significant reductions in depressive symptoms over time. When it comes to current violent behavior, FORNET led to a significantly greater reduction than TAU across all participants. Notably, although individuals with a history of CSA started with higher levels of violent behavior, they showed considerable improvement, reaching similar levels to those without CSA after 6–9 months of treatment. This suggests that while CSA participants initially presented with more severe symptoms, FORNET was effective in reducing violent behavior to levels comparable to those seen in participants without such a history. These findings highlight the broad applicability and effectiveness of FORNET in treating trauma survivors with compounded traumatic experiences, such as CSA. FORNET’s multifaceted approach, which effectively reduces aggression, violent behavior, and PTSD symptoms underscores the importance of combining therapeutic elements, such as the testimony component, which plays a crucial role in the treatment process [14]. The low dropout rates and high retention among participants further suggest strong engagement, highlighting the value of supportive, culturally sensitive therapy [47].

The findings from this sub-analysis highlight the significant impact of CSA on later mental health and social outcomes, reinforcing the importance of early and specific interventions for trauma survivors. FORNET’s success in reducing appetitive aggression, current violent behavior and symptoms of PTSD even among those with CSA histories, underscores its potential as a valuable tool in rehabilitation programs for former combatants and child soldiers. It can make a significant contribution to the success of demobilization and social reintegration of ex-combatants, ultimately reducing gender-based-violence, intimate-parter-violence, as well as family and community violence in post-conflict settings. Our finding of a higher sexual revictimization by superiors among individuals with a history of CSA sheds light on a highly neglected topic - namely sexual abuse and victimization within the military system (military sexual trauma). Considering the young age of first entry into armed groups in this sample, as reported by Koebach et al. [33], with some individuals recruited as early as ages 4 to 12, it is reasonable to assume that at least some instances of the CSA occurred within these armed groups. Therefore, rehabilitation programs for combatants may benefit from incorporating therapeutic tools that aim to reduce the risk of revictimization - for example, by addressing risk recognition, victim mentality, and risky behaviors. Preventive strategies to decrease the risk of further victimization and perpetration, as demonstrated by our sub-analysis, should be treatment target, focusing on adaptive coping and self-protection skills. Specifically, there might be a different therapeutic need in male and female victims; future research should focus on the different gender perspectives of being a victim of CSA and sexual assaults later in life. For instance, women who have experienced CSA may require interventions that address issues related to sexual health and reproductive coercion, while men may need support in dealing with feelings of shame and emasculation [1, 55]. The study also points to the necessity of integrating peacebuilding and stabilization programs with targeted mental health interventions to mitigate the risk of ongoing violence and retraumatization in post-conflict settings, and prevent transgenerational transmission of violence and trauma. Given the high prevalence of residual mental health problems among former combatants, maintaining support networks and promoting positive social bonds are critical for sustainable peace and rehabilitation. In particular, integrating community-based interventions that promote social cohesion and collective healing can help address the broader social determinants of health and prevent the recurrence of violence [5]. In conclusion, FORNET presents a promising therapeutic approach for addressing the complex needs of combat-trauma survivors. The positive outcomes observed in this study advocate for the wider implementation of FORNET in demobilization and rehabilitation programs, ensuring comprehensive support for former combatants and other violent groups. While FORNET proved equally effective for participants with and without a history of CSA, the elevated baseline symptom severity in the CSA group suggests they represent a particularly vulnerable subgroup.

### Limitations

Limitations of the RCT are summarized in Koebach et al. [33]. Regarding our sub-analysis, a significant limitation on the impact of CSA lies in the assessment of CSA itself. Although we obtained significant results, the analyses were based on participants´ subjective reports regarding sexual abuse, and the validity of these were not tested. The specifics regarding the nature and severity of the trauma, such as the frequency, age of occurrence, perpetrators, context (whether it was for sexual desire, humiliation, punishment, or by family members), and other qualitative details were not available. These factors could significantly influence the severity of PTSD, depression, and aggressive behaviors observed later in life. Further research should delve into these aspects to better understand the diverse impacts of CSA [16]. It is well established that the specifics of the trauma experience, such as the relationship to the perpetrator and the context in which the abuse occurred, play a crucial role in the severity of long-term outcomes [26]. Moreover, the high stigmatization of sexual abuse, especially in males, could have led to underreporting and biases in our data. Males are less likely to disclose sexual abuse due to societal stigmas, which can lead to a significant underestimation of its prevalence and impact [30]. This stigma can prevent male survivors from seeking help and fully participating in studies, thereby skewing the results. A recent stigma project highlights that male survivors of sexual violence often face severe stigma, which exacerbates their trauma and complicates their recovery (All Survivors Project, 2017). This possible underreporting might have also affected the depth of the data we could analyze regarding the specific experiences and impacts of CSA and later revictimization during combatant life.

## Conclusion

This study highlights the importance of addressing CSA within treatment settings, as individuals who have experienced such trauma exhibit a complex interplay between early trauma and later mental health and behavioral outcomes, including a higher risk for both victimization and perpetration. FORNET appears particularly effective even for this highly vulnerable group, highlighting its broad applicability. Considering the chronological order of reprocessing traumatic events within NET, it specifically addresses the accumulation of trauma over the lifespan. Therefore, it may be particularly effective for survivors of combat-trauma who have also experienced childhood trauma. Within the demobilization and rehabilitation process, they may benefit from complementary preventive strategies that focus on adaptive coping and self-protection.

In conclusion, FORNET demonstrated its efficacy even in the highly vulnerable group of former combatants with a history of childhood trauma. Ensuring comprehensive and nuanced support for male survivors will be instrumental in fostering sustainable peace and rehabilitation in post-conflict settings.

## Supplementary Information

Supplementary Material 1

## Data Availability

No datasets were generated or analysed during the current study.
