# Supplementary material for: The impact of childhood maltreatment on treatment outcomes for posttraumatic stress symptoms and aggression in male former combatants using narrative exposure therapy [NET] - results from a RCT in Eastern democratic Republic of Congo
Source: Confl Health. 2025 Oct 4;19:67. doi: 10.1186/s13031-025-00710-z (PMC12495712; doi:10.1186/s13031-025-00710-z)
Supplement: Supplementary file 1 — Supplementary Material 1 [file 13031_2025_710_MOESM1_ESM.docx]

**Supplementary Table 1.** PTSD diagnosis by treatment group and CSA after 6-9 months

|  | **TAU** | | **FORNET** | | **Total** | **CSA** | | **Group** | | **CSA x Group** | |
| --- | --- | --- | --- | --- | --- | --- | --- | --- | --- | --- | --- |
| **Variable** | **No CSA (n=194)** | **CSA (n=29)** | **No CSA (n=153)** | **CSA (n=18)** | **Total (n=394)** | $\chi_{\boldsymbol{1}}^{\boldsymbol{2}}$ | **P value** | $\chi_{\boldsymbol{1}}^{\boldsymbol{2}}$ | **P value** | $\chi_{\boldsymbol{1}}^{\boldsymbol{2}}$ | **P value** |
| PTSD Diagnosis 6-9 months, %, n | 48.0, 86 | 59.3, 16 | 30.0, 45 | 22.2, 4 | 40.4, 151 | 0.26 | 0.61 | 16.04 | <.001*** | 0.94 | 0.33 |

*Note:* CSA childhood sexual abuse; P values determined using logistic regression adjusted for age.
